# Development of a culturally adaptable internet-based cognitive behavioral therapy for Japanese women with bulimia nervosa

**DOI:** 10.3389/fpsyt.2022.942936

**Published:** 2022-08-23

**Authors:** Sayo Hamatani, Kazuki Matsumoto, Tomoaki Ishibashi, Ryunosuke Shibukawa, Yuki Honda, Hirotaka Kosaka, Yoshifumi Mizuno, Gerhard Andersson

**Affiliations:** ^1^Research Center for Child Mental Development, University of Fukui, Fukui, Japan; ^2^Division of Developmental Higher Brain Functions, United Graduate School of Child Development, University of Fukui, Fukui, Japan; ^3^Department of Child and Adolescent Psychological Medicine, University of Fukui Hospital, Fukui, Japan; ^4^Research Center for Child Mental Development, Chiba University, Chiba, Japan; ^5^Division of Clinical Psychology, Kagoshima University Hospital, Kagoshima, Japan; ^6^Department of Neuropsychiatry, University of Fukui, Fukui, Japan; ^7^Department of Behavioural Sciences and Learning, Linköping University, Linköping, Sweden; ^8^Department of Clinical Neuroscience, Karolinska Institute, Stockholm, Sweden

**Keywords:** bulimia nervosa (BN), internet-based cognitive behavioral therapy (ICBT), cultural adaptation, cultural sensitivity, ecological validity

## Abstract

**Background:**

The process of cultural adaptation of internet-based cognitive behavioral therapy (ICBT) programs for bulimia nervosa (BN) have rarely been reported despite the potential influence of cultural adaptation of psychosocial interventions on therapeutic response.

**Aim:**

This study aimed to illustrate development process of an ICBT program for Japanese women with bulimia nervosa (BN).

**Methods:**

A mixed methods approach was used to assess cultural adaptation of the prototype of an original ICBT program by using the Cultural Relevance Questionnaire (CRQ). Five women with BN and seven clinicians were interviewed using the CRQ.

**Results:**

Quantitative analyses were conducted to assess cultural adaptation of the prototype of the program and participants rated cultural adaptation as high. A qualitative analysis of the mixed method supported the culturally sensitive changes implemented.

**Conclusions:**

The results of this study show that a series of processes can make ICBT programs more culturally adapted.

## Introduction

### Background

The effectiveness of cognitive behavioral therapy (CBT) for bulimia nervosa (BN) has been demonstrated in multiple clinical trials conducted mainly in Western countries (1). CBT is the therapeutic approach most commonly used to achieve full remission in eating disorders (2). CBT has also been gradually provided in Asia, where the culture is different from the West. Cultural adaption is an important factor in maximizing the potential therapeutic effect of the CBT (3, 4). Cultural adaptation has been defined as “the systematic modification of an evidence-based treatment (or intervention protocol) to consider language, cultural, and context in such a way that it is compatible with the client's cultural patterns, meanings, and values (5)” (6). Culturally adapted interventions are associated with increased remission rates from depressive mood and anxiety symptoms; show odds of 4.68 times higher than other conditions (ie, interventions that do not care for cultural custom) (7). Because culturally compatible psychotherapies potentially may be effective (8), psychologists need to consider cultural sensitivity and ecological validity when developing treatment programs to maximize their potential effectiveness (5).

### Cultural adaptation of CBT

The process of cultural adaptation of CBT treatment manuals or protocols have been rarely documented, including for CBT for BN. A preliminary single-arm clinical trial conducted in Japan suggested the feasibility of CBT for women with BN (9). The results showed that two of seven participants who received the intervention dropped out, and the good treatment outcome was 43.0%. This result appears to be inferior to the therapeutic effect of CBT in Western countries, where the remission rate has been reported to be 54% (10). One potential reason could be the lack of cultural adaptation. The intervention conducted by Hamatani et al. (9) was delivered face-to-face CBT via videoconference using purely Japanese translations of British self-help books (11). The book may have included explanations and tasks that were unfamiliar to Japanese patients.

When providing CBT, differences between Japanese and Western cultures cannot be ignored. For example, Japanese culture tends to avoid assertions in communication and more ambiguous expressions are preferred (12). This indirect communication style can affect the interaction between the therapist and the patient in a CBT session—patients may perceive the therapist as being aggressive when they recommend behavioral changes. In terms of food culture, the staple food in Japan is rice, and “Japanese cuisine (washoku),” which is represented by fermented foods such as natto, pickles, and miso, is unique (13). Establishing a healthy diet is usually included in the treatment module of CBT for BN. During this process, the patient reviews and develops their diet plan (14). Therefore, it is essential to consider dietary culture and communication styles in order for Japanese patients with BN to find the treatment understandable and acceptable.

### Frameworks of cultural adaptation

Several frameworks have been developed for the cultural adaptation of psychotherapies. Existing treatment manuals/protocols are generally used to develop intervention programs for communities with certain cultural characteristics (15–18). This study reports on the cultural adaptation process of an ICBT program for BN. As previously mentioned, the Japanese diet, that is, the food that is the target of fear (target to behavioral experiments or exposure therapy) is quite different from those in the Western culture, where CBT was developed. Therefore, a qualitative study on an ICBT program suitable for Japanese women was conducted. In addition, aiming for cultural adaptation may also be useful when creating an original CBT manual and protocols. The Formative Method for Adapting Psychotherapy (FMAP) is a framework for developing culturally adapted psychotherapy (19). FMAP develops culturally adaptable psychotherapy through the following phases: generating knowledge and collaborating with stakeholders; integrating generated information with theory and empirical and clinical knowledge; reviewing the initial culturally adapted clinical intervention with stakeholders and revising the culturally adapted intervention; testing the culturally adapted intervention; finalizing the culturally adapted intervention.

The Ecological Validity Model (EVM) is often used when cultural adaptations for CBT in a cultural community have been developed (20). Previous studies have considered culturally sensitive aspects based on EVM (language, people, metaphors, content, concepts, goals, methods, context). For example, depression interventions have been developed for Latin adolescents (16), Indonesian individuals (15), Colombian students (17), and Chinese populations (18). These studies suggest that is it feasible to adapt treatments.

### Objective

This paper describes the process of developing Internet-based CBT (ICBT) for BN adapted to Japanese culture. The development of the ICBT program was based on the FMAP approach. We evaluated aspects of EVM when reviewing the prototype of the ICBT program.

## Methods

### Study design

The following three steps were used to develop an ICBT program that is culturally adapted for Japanese women: Phase (a) Develop a prototype of the original ICBT program; Phase (b) Verify the ecological validity of the prototype; Phase (c) Improve the ICBT program. The study describes the above steps and shows the process of developing the culturally adapted ICBT program.

### Phase a: Develop a prototype of the original ICBT program

The first and second authors (SH and KM) designed this ICBT program so that the number of characters, linguistic expressions, concepts, symbols, metaphors, and dietary content would be familiar to Japanese patients. The team has experience of building ICBT programs for other mental disorders—anorexia nervosa (21) and obsessive-compulsive disorder (22). This treatment protocol was designed completely based on a face-to-face CBT manual created by SH and KM.^1^

The treatment modules were influenced by previous studies included the following: psychoeducation and case-formulation (23–29); relaxation and mindfulness meditation (30–34); metacognitive training (35–37); attention training (32); body image modification (37, 38); behavioral experiments against belief about binge-eating impulse (39); establishing healthy eating habits (40, 41); creation of an anxiety hierarchy table and gradual exposure (39, 42); cue exposure to triggers binge-eating and purging (43); cognitive restructuring of negative thoughts (44) re-description of traumatic meaning (45); schema work (46); Prevention of relapse.

As an adaptation/culturally relevant example, a linguistic form is chosen that is natural for Japanese and involves vague nuances in a tone that avoids assertions. For example, “In modern society, even if one leads a careful life in terms of diet, the nutritional value tends to be biased.” In another example, in the case of positive feedback, we used gratitude such as “good work (otsukaresama in Japanese)” and “You did your best (ganbarimashitane in Japanese)” rather than a direct emphasis on the actions they took. Regarding meals, Japanese convenience stores have introduced Japanese recipes. These can be followed even by people who are not good at cooking. Consequently, they do not need to hesitate about what to eat. In addition, in establishing eating habits, we introduce recipes using natto and udon, which are unique to Japan.

### Phase b: Verify the ecological validity of the prototype

We recruited women with BN who were University of Fukui Hospital patients as participants. They were diagnosed with BN by psychiatrists at the Hospital. They met the current or past diagnostic classification of BN based on the Diagnostic and Statistical Manual of Mental Disorders, 5th edition (47). We also recruited clinicians who have treated patients with eating disorders. The participants were asked to evaluate the ecological validity of the prototype for our ICBT program. The protocol was approved by the Research Ethics Committee of University of Fukui in November 2021 (No. 20210136).

To assess the ecological validity of the ICBT program, we used the Cultural Relevance Questionnaire (CRQ), which is a self-report scale used to rate the cultural adaptation of treatment programs (17). CRQ can assess functional, conceptual, and linguistic equivalence. Functional equivalence (items 1–3 of CRQ) can measure whether the behaviors and expressions appearing in the treatment program are familiar to the target population. Conceptual equivalence (item 4 of CRQ) can measure whether the symbols and concepts in the treatment program are universal in the culture. Linguistic equivalence (item 5 of CRQ) can measure language level. We used the CRQ translated in Japanese by the first and second authors of this paper. The respondents rated the five items of the CRQ on a 5-point Likert scale from 1 (Not fit for Japanese culture) to 5 (Perfectly fit for Japanese culture). Other opinions about the program from the participants were collected by the first author (SH).

### Phase c: Improve the ICBT program

The information given by participants as feedback regarding the ICBT program was analyzed, discussed, and incorporated into the second version of the ICBT program by the first and second author (SH and KM).

### Data analysis

We used SPSS Version 26 (IBM Corporation, Armonk, NY, USA) for statistical analyses. First, we reported descriptive statistics on the CRQ to assess the cultural fit of the entire ICBT program and each of its modules. Next, we conducted a qualitative analysis of the comments/opinions and impressions about the ICBT program. We responded to all comments from participants by discussing the revisions made.

## Results

### Phase a

The ICBT program consisted of 12 treatment module and 1 assessment interview module (See Supplementary Figure S1). This ICBT program was created by combining textual and illustrated explanations of each cognitive-behavioral technique with video material and recording sheets. Users can communicate with their therapist to cover their individual treatment plans and homework assignments through the message feature of this ICBT program. The web system that implemented this ICBT program allows users to record their homework every day. Therefore, homework assignments and changes can be done at the user's will and timing. Users can also consult with their therapists using the message tool if necessary. This ICBT program was designed in a way that one module could be completed in about 10–20 min. When converted to a website, it was 4 to 13 pages. The minimum number of characters per page was 36, and the maximum was 1,157. The average number of characters per module was 2,137 characters.

### Phase b

The mean age of the five women with BN who participated in this study was 30.6 (SD = 11.4) years. Four had comorbid mental disorders (2 major depressive disorder, 2 attention-deficit hyperactive disorder, 1 social anxiety disorder, and 1 alcoholism). All participants with BN received pharmacotherapy (3 escitalopram, 2 olanzapine, 1 aripiprazole, 1 mirtazapine, 1 quetiapine, 1 ramelteon). The clinicians included four psychiatrists, two clinical psychologists, and a nurse teacher with a total mean age of 36.1 (SD = 7.5) years. See Table 1 for more information about the participants.

**Table 1:** Characteristics of participants.

	**Women with BN**	**Clinicians**
	**(*n* = 5)**	**(*n* = 7)**
Age, mean (SD)	30.6 (11.4)	36.1 (7.5)
Years of educations, mean (SD)	12.8 (1.1)	19.1 (2.2)
Married, *n* (%)	2 (40.0)	5 (71.4)
Females, *n* (%)	5 (100)	2 (28.6)
Body Mass Index, mean (SD)	19.1 (3.1)	-

For cultural adaptation measured by CRQ, all criteria (functional, conceptual equivalence, linguistic equivalence) were at a high level in both groups. The means of functional, conceptual equivalence and linguistic equivalence for the entire module are as follows: 4.60 (SD = 0.37), 4.60 (SD = 0.55), 4.80 (SD = 0.45) for women with BN respectively; 4.76 (SD = 0.25), 4.86 (SD = 0.38), 4.43 (SD = 0.53) for clinicians. The mean and standard deviation of the CRQ suggest that the ICBT program is culturally fit enough to be accepted by women with BN and clinicians in Japan. There was no significant difference in total CRQ scores between the groups. See Table 2 for more information on the total CRQ score and the difference between the two groups.

**Table 2:** Results of CRQ.

**Item**	**Group**			
	**BN**		**Clinician**	
	**(*****n =*** **5)**		**(*****n =*** **7)**	
	***M***	**SD**	***M***	**SD**
**Entire**				
Functional equivalence	4.60	0.37	4.76	0.25
Conceptual equivalence	4.60	0.55	4.86	0.38
Linguistic equivalence	4.80	0.45	4.43	0.53
**Module 1**				
Functional equivalence	4.53	0.30	4.86	0.26
Conceptual equivalence	4.60	0.55	4.86	0.38
Linguistic equivalence	4.80	0.45	4.14	0.69
**Module 2**				
Functional equivalence	4.60	0.43	4.95	0.13
Conceptual equivalence	4.60	0.55	4.86	0.38
Linguistic equivalence	4.80	0.45	4.43	0.53
**Module 3**				
Functional equivalence	4.73	0.37	4.86	0.38
Conceptual equivalence	4.80	0.45	5.00	0.00
Linguistic equivalence	4.80	0.45	4.71	0.49
**Module 4**				
Functional equivalence	4.60	0.28	4.86	0.26
Conceptual equivalence	4.80	0.45	4.86	0.38
Linguistic equivalence	4.80	0.45	4.71	0.49
**Module 5**				
Functional equivalence	4.87	0.18	4.86	0.26
Conceptual equivalence	4.80	0.45	5.00	0.00
Linguistic equivalence	4.80	0.45	4.86	0.38
**Module 6**				
Functional equivalence	4.80	0.30	4.86	0.38
Conceptual equivalence	4.80	0.45	4.86	0.38
Linguistic equivalence	5.00	0	4.86	0.38
**Module 7**				
Functional equivalence	4.73	0.37	4.95	0.13
Conceptual equivalence	4.80	0.45	4.86	0.38
Linguistic equivalence	5.00	0.00	4.57	0.53
**Module 8**				
Functional equivalence	4.60	0.43	4.90	0.16
Conceptual equivalence	4.80	0.45	4.86	0.38
Linguistic equivalence	4.80	0.45	4.71	0.49
**Module 9**				
Functional equivalence	4.67	0.33	4.95	0.13
Conceptual equivalence	4.80	0.45	4.86	0.38
Linguistic equivalence	4.80	0.45	4.71	0.76
**Module 10**				
Functional equivalence	4.73	0.37	4.95	0.13
Conceptual equivalence	4.80	0.45	5.00	0.00
Linguistic equivalence	4.60	0.55	4.86	0.38
**Module 11**				
Functional equivalence	4.67	0.47	4.86	0.38
Conceptual equivalence	4.80	0.45	5.00	0.00
Linguistic equivalence	4.80	0.45	4.86	0.38
**Module 12**				
Functional equivalence	4.67	0.47	4.91	0.25
Conceptual equivalence	4.80	0.45	4.86	0.38
Linguistic equivalence	4.80	0.45	4.86	0.38

*M, mean; SD, standard deviation*.

### Phase c

The main feedback from the participants was that some sentences were difficult to understand. Thus, we have changed some of the jargon in the prototype of this program to more general terms, in response to comments from participants. For example, “progressive muscle relaxation” was changed to “a method of loosening and relaxing muscles,” and “rumination” was changed to “rumination (negatively, dwell on).” In addition, for the treatment model and cognitive behavioral techniques that were said to be “difficult to understand,” we implemented some videos or photographs of psychoeducation on the module in the ICBT program. Supplementary Figure S2 demonstrates the method of progressive muscle relaxation through textual and visual information in the form of pictures and explains how to perform the relaxation in the order of hands, arms, neck, shoulders, back, legs, abdomen, and face, from the top. Supplementary Figure S3 displays materials on nutritional education, that is, from top to bottom, the percentage of zinc in food (content per 100 g) such as oyster, grilled eel, liver, fried tofu, and almond. More details on comments and responses from the participants, as well as the revised ICBT program can be found in the Supplementary material.

## Discussion

The objective of this study was to develop an original ICBT program for the effective treatment of BN in Japan through a framework of cultural adaptation. An internet-based treatment program was constructed for women with BN to deliver evidence-proven cognitive-behavioral techniques. Five women with BN and seven clinicians evaluated the ICBT program prototype for cultural suitability. Participants provided feedback indicating that the cultural adaptation was consistently high and partially difficult to express linguistically. The developers made various improvements to the program in response to this feedback.

### Primary findings

This study created a prototype of the ICBT program, in which linguistic expressions, including Japanese food in the diet, and daily life including eating habits, were adapted to Japanese culture. The developers paid attention to the ecological validity of the ICBT program (5). The results of evaluating cultural adaptation using the CRQ showed that the prototype of the ICBT program developed now is adapted for Japanese culture based on the participants' evaluation. Therefore, our results may suggest that the EVM is important even in the initial development process of an ICBT program. To date, internet intervention programs have rarely if ever used cultural adaptation methods (17). The development process described in this study contains useful information for researchers in this field. To the best of our knowledge, this ICBT program is the first to use the CRQ to develop an original culturally adaptive intervention on Japanese culture.

### Process of developing the culturally adaptive ICBT program

The ICBT program was designed so that participants can complete one module in 10–20 min. In terms of character volume, the average number of characters per 1 module was 2,137 characters in Japanese (When Japanese is translated into English, the number of words in English is about half compared to Japanese). Previous research reported that the average length of the written text for the session was 1,711–3,000 words (48, 49). Therefore, there may be a little less information in our program. However, almost all the participants with BN (*n* = 4 of 5) commented, “This is an appropriate amount of information.” Furthermore, as three out of five patients with BN commented “If there is more information than this, it is too much information.” Thus, we retained the amount of information as is. ICBT for BN is reported to have a relatively high dropout rate (50, 51). Therefore, this amount may be just right and acceptable for Japanese patients with BN to prevent dropouts. In addition, manga is representative of Japanese culture (52). As shown in Supplementary Figure 2, Japanese people prefer to see both illustrations and letters simultaneously. This preference may be influenced by the Japanese manga culture. A clinician also commented that “photographs of foods rich in nutrients should help patients with BN imagine one.” The developers have added some pictures of nutrient-rich foods in response to this comment. This supports the importance of involving clinicians as well as patients in designing culturally adapted psychotherapy (19).

### Limitations

First, due to the small number of participants in this study, our findings may not represent the entire population with BN. Of the five patients in this study, four had relevant mental/addiction comorbidities, and all participants with BN received pharmacotherapy. Thus, the participants might be women with relatively serious BN cooperated. Another study is probably needed to determine whether our ICBT program is culturally adaptable to populations whose symptoms are not as severe. Further research is recommended to understand better whether cultural adaptation is beneficial for patients with BN. Second, the CRQ Japanese version as the assessment tool of cultural adaptation was not standardized. Third, this study did not include data on participants who had been exposed to the intervention, making it impossible to assess the effectiveness of the culturally adaptive ICBT program developed. In the near future, we will investigate the feasibility and effectiveness of our ICBT program in a randomized controlled trial.

## Conclusions

In conclusion, this study illustrates via a series of processes the development of a culturally adapted ICBT program. The ICBT program may be adequately adapted for Japanese patients. However, more research is needed on the feasibility and effectiveness of this program by conducting a pilot study, preferably as a prospective randomized controlled trial.

## Data availability statement

The raw data supporting the conclusions of this article will be made available by the authors, without undue reservation.

## Ethics statement

The protocol of this phase was approved by the Research Ethics Committee of the University of Fukui in November 2021 (No. 20210136). The patients/participants provided their written informed consent to participate in this study.

## Author contributions

Conceptualization and methodology: SH, KM, and GA. Formal analysis and funding acquisition: SH. Investigation: SH, KM, TI, RS, YH, HK, and YM. Project administration and writing—original draft: SH and KM. Supervision: YM and GA. Writing—review and editing: SH, KM, TI, RS, YH, HK, YM, and GA. All authors contributed to the article and approved the submitted version.

## Funding

This work was supported by the Grants-in-Aid for Scientific Research from the Japan Society for the Promotion of Science (JSPS) (Grant Numbers 18K17313, 19J00227, and 22H00985).

## Conflict of interest

The authors declare that the research was conducted in the absence of any commercial or financial relationships that could be construed as a potential conflict of interest.

## Publisher's note

All claims expressed in this article are solely those of the authors and do not necessarily represent those of their affiliated organizations, or those of the publisher, the editors and the reviewers. Any product that may be evaluated in this article, or claim that may be made by its manufacturer, is not guaranteed or endorsed by the publisher.

## Footnotes

^1^Hamatani S, Matsumoto K. *A Manual of Cognitive Behavioral Therapy for Blimia Neruvosa—Therapsit Version*.
