# Supplementary material for: Development of a culturally adaptable internet-based cognitive behavioral therapy for Japanese women with bulimia nervosa
Source: Front Psychiatry. 2022 Aug 23;13:942936. doi: 10.3389/fpsyt.2022.942936 (PMC9446753; doi:10.3389/fpsyt.2022.942936)
Supplement: Supplementary file 1 [file Data_Sheet_1.docx]

**Supplementary File**

**Development of a culturally adaptable internet-based cognitive behavioral therapy for Japanese women with bulimia nervosa**





FigureS1. Home screen of the assessment and treatment module

FigureS2. Adaptation by adding some photographs to the method of progressive muscle relaxation

**
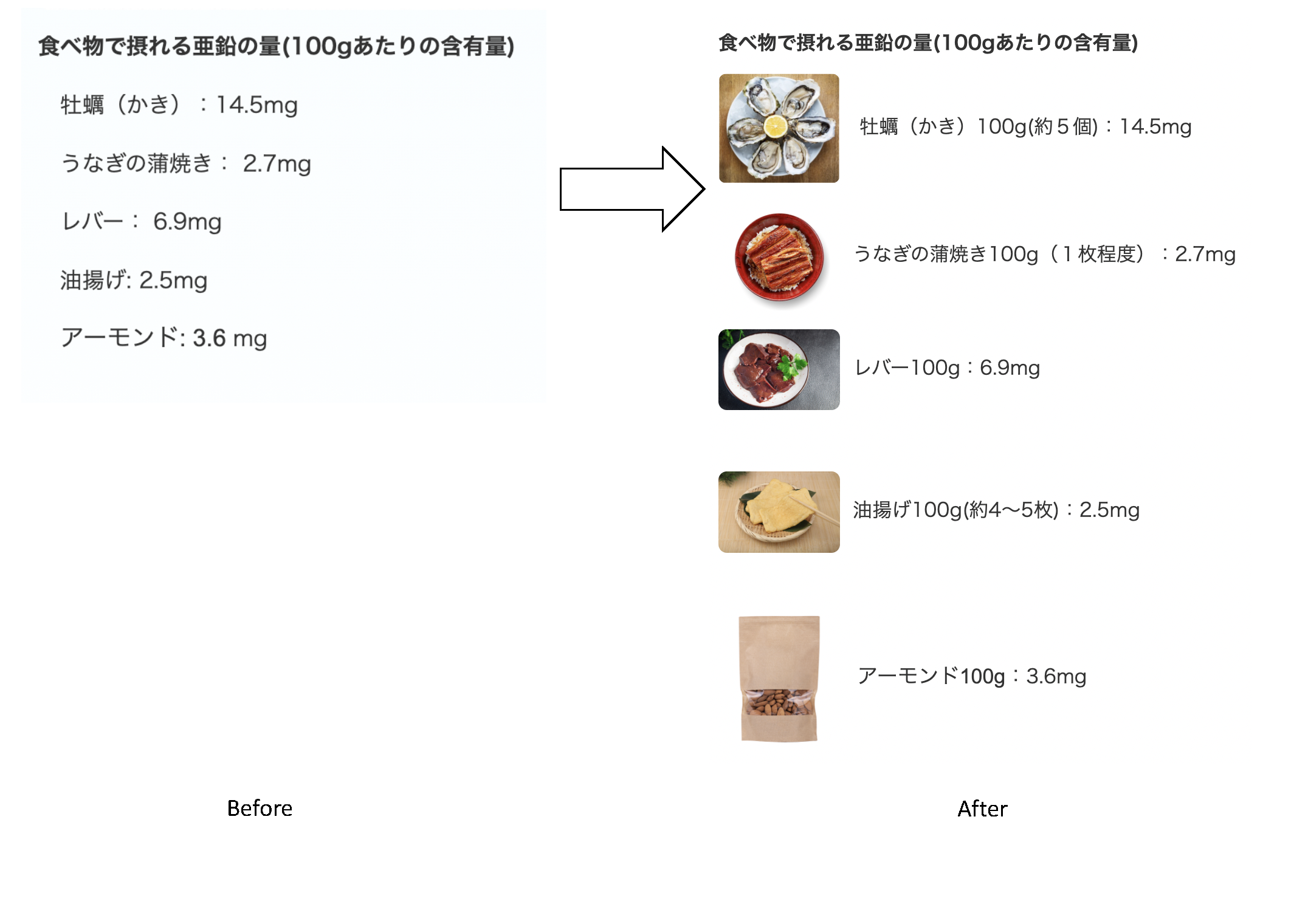
**

FigureS3.　Adaptation by adding some photographs in module 6

Table S1. Categories and Examples of Cultural Adaptations

| Dimension | Example (before adaptation) | Issue | Adaptation decision | Example (after adaptation) |
| --- | --- | --- | --- | --- |
| Language | "Vomiting," "self-induced vomiting" | Confused since there are multiple terms | Unified terms | "Vomiting" |
|  | "The problem begins after a strict diet." | It is unclear what the problem refers to | Clarified the expression | "Overeating and vomiting begins after a strict diet." |
|  | "Bulimia nervosa is a mental illness characterized by anxiety and extreme immersion in weight and body shape, as well as weight gain or fear of weight gain." | The expression is redundant | Clarified the sentence | "Bulimia nervosa is a mental illness characterized by a strong fear of weight gain due to severe weight and body shape concerns." |
|  | Extreme immersion in body shape | Difficult to understand | Made the expression easier to understand | "To be terribly concerned about body shape and weight." |
|  | "Prevalence" | Language is difficult | Added explanation | Prevalence is the percentage of individuals who have a disease at a given time. |
|  | "Telepathy" | Language is difficult | Added that there is an explanation later | "I will explain each term later." |
|  | "Narrowness about society (narrow view of things)" | Language is difficult | Removed some terms | "The view of things is narrow" |
|  | "Non-functional eating habits" | Difficult to understand | Changed the term | "Irregular eating habits" |
|  | Physiological response described in the procedure of cognitive behavior model. | Difficult to understand | Deleted | N/A |
|  | Case formulation | Language is difficult | Added explanation | "Case formulation (method for understanding the vicious circle)" |
|  | Maladaptive beliefs | Language is difficult | Added explanation | Maladaptive beliefs (negative firm beliefs) |
|  | Systemic muscle relaxation method | Language is difficult | Changed to a simple term | "How to relax muscle stiffness" |
|  | "Dissatisfaction with body image" | Inappropriate terminology | Changed to an appropriate term | "Body image distortion" |
|  | "Feet are short" instead of "feet are ugly" was stated as an example of objective evaluation. | "Feet are short" may provide a negative impression | “Feet are short" has a less negative impression than "feet are ugly." Since it is more objective than the average value, whether the legs were short or long, we did not change them. | N/A |
|  | QOL | QOL may be an unfamiliar abbreviation. | Added explanation | QOL (Quality of life in Japanese) |
|  | "Eating disorder" | It is confusing because there are several types of eating disorders | Changed the term | "Binge eating" |
|  | Examples of metacognition are not written. | Difficult to imagine | Added each example | Explanation of telepathy "The woman laughing at me thinks my body is fat and she must be mocking me." |
|  | Rumination | Language is difficult | Added explanation | "Rumination (negatively, dwell on)" |
|  | "If there is something scary to eat" | Difficult to read | Clarified | Food that makes you feel uneasy and scared. |
|  | "One banana," "One chocolate" | Difficult to read since the units are not unified. | Described the unit accurately | "a piece of banana," "a piece of chocolate" |
|  | Rebellion | Difficult to imagine | Changed the term | Resistance |
|  | Ruin of life | Expression is too strong | Deleted, judging that it is not a term used for people with bulimia nervosa. | N/A |
|  | "Traumatic stress disorder" | Not an official name | Corrected the term | "Post-traumatic stress disorder" |
|  | "A person who had a very painful experience as a child, some people suffer from bulimia nervosa." | "Some people who suffer from bulimia had a very painful experience as a child." This text is more natural. | The change was not made. The inversion method was used to emphasize the text. | N/A |
|  | "Rewriting the meaning of traumatic memory." | The word "meaning" is confusing. | Deleted | "Rewriting traumatic memory" |
|  | "Vulnerability (vicious circle)" | The word "vulnerable" is difficult to understand. | Deleted since the meaning can be conveyed without the word vulnerable | Vicious circle |
| Methods | Eating disorders can be broadly divided into three categories. ・Anorexia Nervosa Underweight and commonly known as anorexia nervosa. ・Bulimia Nervosa hyperphagia | It is difficult to see at a glance. | Added a serial number | Eating disorders can be broadly divided into three categories. ① Anorexia Nervosa Underweight and commonly known as anorexia nervosa. ② Bulimia Nervosa hyperphagia |
|  | Cognitive-behavioral therapy is what happens in the mind (cognition), such as perception, attention, image, emotion, memory, and thinking… | Difficult to read | Changed the position of the word | Cognitive-behavioral therapy is cognition (what happens in the mind: perception, attention, image, emotion, memory, thinking, etc.) and … |
|  | Describe a case formulation using sentences only. | Difficult to create | Added video | N/A |
|  | It may be better to change the title to “Autonomic nerves.” | The term autonomic nerves appear suddenly. | The title was not changed since we decided that it was not desirable to introduce relaxation techniques without discussing autonomic nerves. | N/A |
|  | The explanation of the breathing method. 1. First, sit comfortably on the chair. 2. Inhale naturally from your nose in 3 seconds. | Difficult to imagine only by reading a description | Added video | N/A |
|  | The first explanation of the breathing method does not indicate when to do it. | Do not know immediately when to do it | This module explains what to do when you feel stressed. It is possible that this participant simply missed this information. The ICBT program was not amended. The homework specifies when and how often relaxation techniques such as breathing should be done. | N/A |
|  | The explanation of the behavioral experiment method is only in sentences. | It is difficult to understand how to record | Added video | N/A |
|  | Conducting behavioral experiments | Unfamiliar words | The term "behavioral experiment" is retained to describe a therapeutic technique with a specific purpose. In addition to videos that help understanding behavioral experiments, we introduced the behaviors using suitable examples for the Japanese culture. | N/A |
|  | The explanation for negative automatic thinking is only in the text. | Confusing | Added video | N/A |
|  | Text of the introduction of exercise: “Let’s rewrite the traumatic memory in the following steps.” | Unnecessary | Not changed, since it is easier to understand what you are going to do. | N/A |
| Content | Performing mindfulness meditation for 15 minutes. | 15 minutes of meditation time is too long for some individuals. | Not changed, since it does not force you to meditate for 15 minutes; however, added description. | "Let us work on mindfulness meditation for 15 minutes. However, if you do not have the time. Let us begin with 5 minutes" |
|  | Performing breathing exercises in the morning, noon, and night. | Working individuals may not be able to do breathing exercises during the day. | Not changed, the breathing method is 3 minutes and is relatively easy to do. | N/A |
|  | Total number of sessions (Module 6) | Patients may not read due to the large amount of information | Since the amount of information is large, we added a video with a supplementary explanation that you should read it gradually. | N/A |
|  | The weight of the ingredients is written in grams. | It is difficult to imagine the specific amount. | Added some photographs. | Figure 2. |
|  | Handling of anxiety during exposure tasks. "You do not have to do anything after overeating. Let us wait for your anxiety to diminish." | Difficult to wait without nothing. | Added explanation. | "After overeating, do your usual activities, such as watching TV, watching videos, playing games, or just sitting back and relaxing in your chair. Feel less anxious!" |
|  | Assessing the intensity of anxiety 60 minutes after exposure | It may be better to describe the intensity of anxiety at 10, 20, and 60 minutes after exposure. | Not changed, since there is a risk of discontinuing the exposure after 10 and 20 minutes of evaluation, recognizing that the anxiety has not decreased. The key factor is the assessment after 60 minutes. | N/A |
|  | Read the contents of the trauma aloud as homework every day. | May be very painful for the patient. | Did not change reading aloud every day. However, a video has been added to enable the participant to continue. | N/A |
|  | Negative spiral | Expression may be too strong. | Not changed, since expressions such as "from there" are ambiguous and clarifying them promotes understanding. | N/A |
|  | Safe behavior | Impression that the word “safe behavior” suddenly appears. | In the explanation of the behavioral experiment, the word "safety behavior" was not used. Therefore, it was deleted in recurrence prevention. | N/A |
|  | As for homework, only the homework of the session at that time is presented. | It is better to encourage reviewing the program to remember | Not changed. With each session, the amount of homework increases, the patient becomes disgusted, and the risk of interruption increases. | N/A |
| Context | "By taking a slow and deep breath, you can work on the parasympathetic nerves. In other words, by repeating a slow and deep breath, you can relieve the tension in your body." | The expression was redundant. | Removed duplicates for brevity. | "By breathing slowly and deeply, you can work on the parasympathetic nerves, which means that you can relieve tension in your body." |
|  | It is important for a female’s nutritional health to consume 2000, 2050, and 1950 kcal during her 20s, 30s and 40s, and 50, respectively). | It is difficult to understand since it is written according to age. The required calorie intake does not differ significantly from 20 to 50 years of age. It is easier to understand if they are unified. | Described together | "A nutritional intake of 2000 kcal is recommended for a female, since it does not significantly change according to age." |
| People | "I want to take laxatives when I see a thin person.” | The patient may not experience the exact same urge. | Changed emotional expression. | "I think I have to lose weight when I see a thin person" |
|  | The names of all the characters are "Mei (a Japanese girl name)." | It is confusing if the same name is used in different settings. | Used different names for each setting. | "Mei," "Chihiro," "Satsuki," etc. |
| Metaphors | "trigger” | Patients may not understand the example of "trigger." | Changed to another word | “Cue” |
| Goals | N/A |  |  |  |
| Concept | N/A |  |  |  |
